# Methodological challenges in studying disease processes using observational cohort data

**DOI:** 10.1007/s42081-024-00276-9

**Published:** 2024-10-30

**Authors:** Richard J. Cook, Jerald F. Lawless

**Affiliations:** Department of Statistics and Actuarial Science, University of Waterloo, Waterloo, ON N2L 3G1 Canada

**Keywords:** Dynamic processes, Intervention effects, Longitudinal studies, Multistate models

## Abstract

Cohort studies of disease processes deal with events and other outcomes that may occur in individuals following disease onset. The particular goals are often the evaluation of interventions and estimation of the effects of risk factors that may affect the disease course. Models and methods of event history analysis and longitudinal data analysis provide tools for understanding disease processes, but there are numerous challenges in practice. These are related to the complexity of the disease processes and to the difficulty of recruiting representative individuals and acquiring detailed longitudinal data on their disease course. Our objectives here are to describe some of these challenges and to review methods of addressing them. We emphasize the appeal of multistate models as a framework for understanding both disease processes and the processes governing recruitment of individuals for cohort studies and the collection of data. The use of other observational data sources in order to enhance model fitting and analysis is discussed.

## Introduction

Much of modern medical research is concerned with the study of disease processes and their effective management. Most disease processes are complex and both affected individuals and the factors that influence their disease are heterogeneous and often incompletely understood. Longitudinal cohort studies and other types of observational data are critically needed to understand such processes; studies that regularly collect detailed and accurate data on individuals over an extended period of time are especially valuable. Such studies are also a source of what has been labelled real world data (RWD) and real world evidence (RWE) on the effectiveness of interventions in broad patient populations. We address such studies here, along with other sources that provide RWD.

We consider contexts where the scientific objectives are to understand the dynamics of disease processes, factors that affect them, and the effectiveness of disease management (i.e. treatment) strategies. The term dynamics refers to the ways that diseases and their treatment evolve and affect an individual over time. We have argued elsewhere that stochastic event history models, and in particular, multistate models, provide an important and powerful framework for studying disease in cohort studies (Cook and Lawless, 2018, 2022) and in randomized trials (Bühler et al., 2023; Cook and Lawless, 2024a). We consider disease processes that can be usefully represented by such multistate models.

Our objectives are to describe the challenges in modeling and analyzing observational data from disease cohort studies and to review methods for addressing them. We will describe the ancillary role of multistate models in addressing challenges related to the collection of data in observational studies and discuss forms of auxiliary data that can mitigate the effects of non-ignorable data collection processes. Methods of assessing the effects of interventions in complex settings will be outlined.

Data from research studies on rheumatic disease conducted at the Centre for Prognosis Studies in the Rheumatic Diseases at University of Toronto (Gladman and Chandran, 2011) will be used to discuss and illustrate issues we consider. One such study is based on the University of Toronto Psoriatic Arthritis Cohort (UTPAC), consisting of individuals with psoriatic arthritis (PsA). This registry was established in 1978 and currently has about 1700 members who are followed and studied in order to understand the dynamics, progression and treatment of PsA. Related research involving rheumatic diseases includes registries of patients with lupus (Gladman et al., 2002), psoriasis (Eder et al., 2011), and other immunological conditions.

The remainder of the paper is organized as follows. Section 2 reviews disease processes, multistate models and cohort studies. In Sect. 3 we discuss challenges in recruiting cohort members and in collecting accurate data on their disease processes. We stress the importance of considering the conditions leading to selection, how dependent delayed entry and left-truncation can bias inferences, and show how multistate models can provide a framework for addressing these challenges. Section 4 describes some other sources of information on disease processes, including registries, surveys and electronic health records, and how they can supplement analysis of cohort studies. Section 5 considers the assessment of interventions, based on observational data. Section 6 illustrates issues discussed in the context of psoriatic arthritis and the UTPAC. Section 7 concludes with comments on the collection of high quality observational data, and on areas where research is needed.

## Disease processes, multistate models and cohort studies

### Disease processes and multistate models

**Fig. 1:**
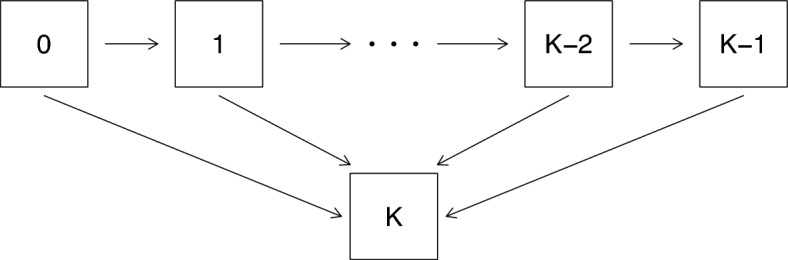
A progressive disease process with a terminal death state

Consider disease processes where an individual is in one of a set of defined states at a time *t* following disease onset; such processes can be represented by multistate models. We consider processes that have transient states $\{0, 1, \ldots , K-1 \}$ and an absorbing state *K*, which we take to represent death. Such processes cover a wide range of settings including failure time or disease progression modeling, illness-death processes, and recurrent and terminal events. Let *Z*(*t*) denote the state occupied at time $t \ge 0$ for a given individual, where $t=0$ is the time of disease diagnosis; when viewed as a stochastic process we write $\{Z(s), 0 \le s\}$. Figure 1 depicts a process often used to model progressive diseases. For the general population, state 0 could represent absence of disease, with state 1 entered upon disease onset; here *t* would represent age. If the study population consists instead of just persons with the disease, state 0 could represent the lowest stage of the disease, with states $1, \ldots , K-1$ representing increasing levels of disease burden. In this case *t* could be the time since disease onset. The case $K = 2$ is the much-used illness-death process (Cook and Lawless, 2018); for example, entry to state 1 might represent the onset of diabetes or some form of cancer in disease incidence studies. For studies of persons treated for a form of cancer, state 0 might represent a disease-free state following treatment, and state 1 a cancer recurrence state. In research on aging and cognition we might have $K = 4$, with state 0 representing full cognition and states 1, 2 and 3 representing mild, moderate and severe cognitive impairment (Lawless, 2013). Cook and Lawless (2018) discuss multistate processes at length, and provide many examples of their application.

In many settings there may be fixed or time-varying covariates *V*(*t*) reflecting aspects of health or disease activity; though some elements of *V*(*t*) could be fixed we denote the covariate process by $\{V(s), 0 \le s\}$ for generality. We consider continuous-time models, which are specified by their transition intensity functions; see Chapter 2 of Andersen et al. (1993) and Section 1.3 of Cook and Lawless (2018). The $k \rightarrow r$ transition intensity is

2.1 $$\begin{aligned} \lambda _{k r}(t | {H}(t)) = \lim _{\Delta t \downarrow 0} \dfrac{P(Z(t + \Delta t) = r | Z(t) = k, {H}(t))}{\Delta t},~ k \ne r \end{aligned}$$

where ${H}(t) = \{ Z(s), V(s), 0 \le s < t\}$ is the complete process history up to time *t*, including observed covariate values. We let $N_{k r}(t)$ denote the number of *k* to *r* transitions over $(0, t], k \ne r$, and let *N*(*t*) be the vector of all such transition counts over (0, *t*]; we also let $d N_{kr}(t)$ and *dN*(*t*) indicate particular transition events at time *t* (e.g. Aalen et al., 2008). With this notation we write ${H}(t) = \{d N(s), 0< s<t, Z(0), V(s), 0 \le s < t\}$; typically $P(Z(0)=0)=1$.

Intensities describe the dynamic properties of a process through the instantaneous probabilities of transitions from one state to another at a given time; under mild conditions they fully specify a multistate process mathematically when only fixed covariates *V* are present. We defer a discussion of time-varying covariates until later. Intensities are conditional in the sense that at time *t* they depend on an individual’s past history *H*(*t*). Certain families of processes relax these conditions. In particular, Markov processes assume that transition intensities are a function of *t* and covariates only; see Section 2.3 of Cook and Lawless (2018). Based on the transition intensities for a process, we can compute other features of interest. Key features include transition probabilities $P(Z(t+s)=r|Z(t)= k, {H}(t))$ and state entry probabilities $P(T_k \le t|{H}(0))$, where $T_k$ denotes the time of entry to state *k*.

### Cohort studies

Sources of information on disease processes for individuals include randomized trials, observational cohort studies, registries that include information on persons with a specific disease, and administrative data found in electronic medical records. Randomized trials provide high quality information on treatment or intervention effects on a disease, but are usually conducted on a narrowly defined patient population and under more tightly controlled conditions than in the broader population of affected individuals. There is consequently much interest in so-called “real world” data (RWD) on disease processes and interventions, such as can be found in observational studies, registries and administrative records (Crown, 2019; Klonoff, 2020; Togo & Yonemoto, 2022). Such sources of data come with limitations, however, and if these are ignored, can provide biased estimates of disease process features. This is the main topic of the present paper. Our main focus will first be on disease cohort studies, followed by discussion of some other types of observational data on disease processes.

Important limitations of many cohort studies (and other observational data sources) are related to how individuals are selected for a study, and to how data on individuals are collected over time. In particular, individuals recruited for a study may not be a representative sample of persons in a perceived study population. In addition, data on individuals who join a study are often collected at random individual-specific time points, such as at clinic visits, periodic interviews, or online at certain followup times. These observation times and the accuracy of the data collected may depend on a person’s disease history and personal characteristics. We introduce two cohorts associated with rheumatic diseases that we use later to illustrate points discussed in the paper. Other areas in which multistate models have proven useful include heart disease (Ieva et al., 2017), aging research, cancer and diabetes (Cook and Lawless, 2018).

*Illustration: University of Toronto Psoriasis and Psoriatic Arthritis Cohorts*

Psoriatic arthritis (PsA) is a progressive inflammatory disease that can lead to swelling and damage in a person’s joints, and may result in functional impairment and disability (Gladman & Chandran, 2011). The University of Toronto Psoriatic Arthritis Cohort (UTPAC) was established in 1976 to study affected persons and continues to recruit and follow individuals with PsA. It currently has about 1700 members and has been a major source of information on the disease process, rates of progression and therapies for the condition. Most persons with PsA have previously been diagnosed with psoriasis (Ps), which is a chronic condition involving plaques and lesions on the skin (Eder et al., 2019); about one-third of persons with Ps develop psoriatic arthritis. In 2008 the University of Toronto Psoriasis Cohort (UTPC) was established; it currently has about 700 members.

## Models for disease processes, cohort selection and data collection

### Cohort recruitment

We consider here the assembly of a cohort for the purpose of studying disease processes for a specific study population of affected persons; we focus on prospective studies in which cohort members are followed over some time period following enrollment. Recruitment of participants for such cohorts occurs in a variety of ways, but a common issue is that the likelihood a person in the study population enrols in the cohort depends on their disease history and on personal characteristics. It is often useful to distinguish between what we refer to as type A and type B studies (Cook and Lawless, 2024b). A type A study is one in which cohort members are chosen through random sampling of persons in the study population, whereas a type B study is one in which individuals join a cohort at least in part through their own actions in seeking care or medical treatment. Large scale longitudinal health surveys provide examples of type A studies, but they typically are not focussed on a specific disease process; we discuss health surveys in Sect. 4. We focus here on the more common type B studies.

The University of Toronto Psoriatic Arthritis Cohort (UTPAC) described in the preceding section is a type B cohort. It targets persons who have been diagnosed with psoriatic arthritis (PsA), but both enrolment in the cohort and the length of time until an affected person enrols may depend on their age and sex, pattern of medical care (primary care physicians are for example more likely to refer an affected person to the clinic overseeing the cohort) and on the extent to which they experience symptoms such as pain or swelling in joints. Some affected persons may never enroll in the cohort, especially if their disease does not progress much or if they are receiving adequate medical care. The “study population” is usually ambiguous in settings like this because even if a geographic region and other conditions for enrolment have been specified, the number of affected persons in the population at any given time is typically unknown. This is the case for UTPAC since a complete registry of persons diagnosed with PsA does not exist. In such cases the study population is nevertheless a useful concept, especially when considering the generalizability of results from analyses of cohort data. There has been much recent discussion about this, along with the related concept of transportability of research findings (Degtiar & Rose, 2023; Keiding & Louis, 2016), which refers to whether study results apply to some population or group. Slightly different definitions can be given, but here we take generalizability and transportability to mean that estimates of disease processes and their features based on a study are (reasonably) accurate estimates of the same things in some population. Cook and Lawless (2024c) provide a recent review of these and other concepts for rheumatologists.

Standard methods of analyzing event history data from a cohort are based on conditional independence assumptions concerning entry to a study and the process intensities following entry (or enrolment). For discussion, suppose that the time origin for the disease process of interest is $t=0$ and that a given person in the study population enrols in the cohort at time $L \ge 0$. The enrolment conditions require that the disease process $\{Z(s), L< s\}$ for this person during followup in the cohort is representative of those in the study population, and having the same disease history *H*(*L*) as the cohort member. These conditions can easily be violated. For example, persons enrolling in the cohort may form a biased sample of persons in the study population, due to unobserved (and therefore unaccounted for) factors that affect both enrolment and the disease process. There may also be different environments in the cohort and the population at large which affect the disease process. We have recently discussed independent entry conditions in detail (Cook and Lawless, 2024b), and point out that unless some direct comparison is possible of persons in and outside a cohort, these conditions cannot be guaranteed.

We have elsewhere (Cook & Lawless, 2021, 2024b) stressed the importance of modeling cohort enrolment and data collection processes jointly with the disease process of interest. This facilitates study of potential sources of bias in cohort data analysis as well as the transportability of study results. We have also noted (Cook & Lawless, 2024b) the necessity of auxiliary data on the study population in order to do this. We will discuss this more fully in Sect. 4, where we consider information on disease processes that is available in some observational data sources. We focus discussion in the remainder of this section on cohort recruitment; often it is something that researchers cannot control well.

To examine recruitment rigorously we formulate a joint model for enrolment and the process of interest. We let *L* denote the potential time of entry to the study for a given individual in the study population, and let $Y(t) = I(L \le t)$ indicate whether the individual has entered the study by time *t*. We then let $\mathcal{Z}(t) = (Z(t), Y(t))$ record the disease state and recruitment status of an individual and $\{{{\mathcal {Z}}}(s), 0 \le s \}$ denote the joint process with history ${{\mathcal {H}}}(t) = \{ {{\mathcal {Z}}}(s), 0 \le s < t, V \}$. We continue for now to consider only fixed covariates *V* observed at time of entry. Recruitment or study entry is said to be conditionally independent of the multistate process if for $t > 0$

3.1 $$\begin{aligned} P(d N(t) | {{\mathcal {H}}}(t)) = P( d N(t) | H(t)). \end{aligned}$$

This is consistent with the way that independent delayed entry and left-truncation (and also right-censoring) is defined in event history analysis; see Section 2.2.8 of Aalen et al. (2008) and Section 2.1 of Lawless and Cook (2019). Note that (3.1) implies that the transition intensities for a person in the study at time *t* are the same as the intensities for a person with the same process history who is in the population but not in the study. This is why independent delayed entry is necessary for study findings to be generalizable.

We note that conditioning on the study entry time $L=l$ and on the observed history at time *l* is crucial for analysis of outcomes at times $t>l$. Here, $L=l$ is a left-truncation time when referring to the sojourn in the state occupied at time *l*. Various authors discuss bias that may arise when this is ignored. For example, Tamura (2023) consider so-called “length bias” in the analysis of failure times for cancer patients among persons undergoing genomic profiling in Japan.

**Fig. 2:**
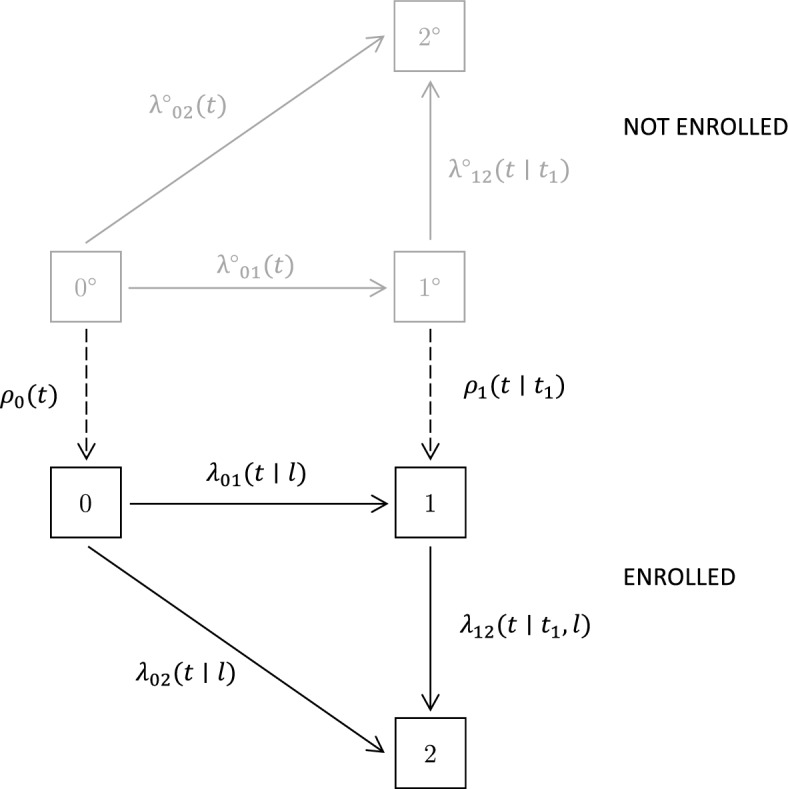
A joint model for an illness-death and enrolment process

Recruitment and enrolment in a cohort can be examined by jointly modeling the disease process and the study recruitment process. A type B study for an illness-death process is illustrated in Fig. 2. This differentiates disease states according to whether an individual has or has not been enrolled in the cohort. Here $\rho _0(t)$ and $\rho _1(t|t_1)$ are the study enrolment intensities for persons in state $0^{\circ }$ and state $1^{\circ }$ respectively. We let $t_1$ denote the time of a transition from state $0^{\circ }$ to state $1^{\circ }$ or from state 0 to state 1. In our notation *Y*(*t*) switches from 0 to 1 at enrolment; for Fig. 2 this occurs when a transition is made from state $0^{\circ }$ to state 0 or from state $1^{\circ }$ to state 1. The independent study recruitment condition (3.1) fails to hold when the intensities for enrolled and unenrolled individuals are different. We allow for this by denoting process transition intensities before enrolment as $\lambda ^{\circ }_{01}(t),\lambda ^{\circ }_{02}(t)$ and $\lambda ^{\circ }_{12}(t|t_1)$ and those after enrolment as $\lambda _{01}(t|l),\lambda _{02}(t|l)$ and $\lambda _{12}(t|t_1,l)$. Note that intensities after enrolment may depend on the time *l* of enrolment.

We now consider likelihood functions based on observed data for a cohort study. We continue to restrict attention to fixed covariates *V*. For a type B enrolment process, suppose that for an individual who has enrolled at time $L=l$, process history *H*(*l*) is ascertainable. The full data on such an individual then consists of the time *l*, the state *Z*(*l*) and prior disease history plus data observed during subsequent followup in the cohort. The likelihood contribution based on these data then takes the form (ignoring censoring for simplicity) $L_F = L_{R}L_{P}$ where

3.2 $$\begin{aligned} L_{R}&= P(L = l,{Z}(l), {{\mathcal {H}}}(l) | {{\mathcal {Z}}}(0) = (0,0), V, \Delta = 1) \end{aligned}$$

3.3 $$\begin{aligned} L_{P}&= P( {Z}(s), l < s | L=l, {Z}(l), {{\mathcal {H}}}(l)) \end{aligned}$$

and $\Delta$ indicates enrolment in the cohort. The condition $\Delta = 1$ for type B enrolment implicitly includes the condition $L \le L^{\dag }$, where $L^{\dag }$ is the upper limit on the entry time for that individual in the population, determined by the recruitment period for the study. The parts $L_R$ and $L_P$ of the likelihood are respectively termed the retrospective and prospective components. It is implicit in (3.3) that $\Delta =1$; this is suppressed notationally for simplicity.

Although it is not our main focus, we note that for a cohort with type A enrolment, an individual is (randomly) selected at a specified time $L=l$. In this case the likelihood $L_P$ remains the same but $L_R$ becomes

3.4 $$\begin{aligned} L_{R}&= P({Z}(l), {H}(l) | L=l, {Z}(0) = 0, \Delta = 1) . \end{aligned}$$

Section 7.1 of Cook and Lawless (2018) gives these expressions in a discussion of state-dependent selection under the assumption that selection satisfies the independence condition (3.1). They observe that unless sufficiently detailed information on the multistate process history prior to entry is ascertainable, $L_{R}$ will not typically be useable. For the illness-death process this would include the time $t_1$ of a transition from state $0^{\circ }$ to state $1^{\circ }$ and covariates *V*. The minimum requirement for within-study inference based on the prospective likelihood $L_{P}$ is that the history ${{\mathcal {H}}}(l)$ needed to compute (3.3) be available. Under the independence condition (3.1) contributions to $L_{P}$ are expressed in terms of the intensities in (2.1) and inferences apply to the study population. In practice there may be incomplete information about the process prior to cohort entry and we typically replace (3.3) with

3.5 $$\begin{aligned} L_{P}^{{\textrm{obs}}} = P(Z(s), l < s| L=l, H^{\textrm{obs}}(l)), \end{aligned}$$

where ${H}^{{\textrm{obs}}}(l)$ denotes the available (“observable”) information about *H*(*l*). This often leads to the adoption of models for which (3.5) is the same as (3.3). Markov models where $\lambda _{k r}(t | {H}(t)) = \lambda _{k r}(t|V)$ are one such example since $H^{\textrm{obs}}(l) = (Z(l^-),V)$ is sufficient for specification of the intensities. We note that for processes with time-varying covariates *V*(*t*) there is usually no information on *V*(*t*) for $t < L$.

### Observation processes and data collection

Followup data on cohort members is typically collected at intermittent times, often during visits to a care or assessment facility. If these visits take place according to a schedule, the main issue is that information on the disease process between visits is unobservable. A more problematic scenario is when visits occur randomly and are related to a person’s medical time-varying markers (covariates) or disease process. If the visit times are improperly treated as ignorable, then estimates of disease features will be biased. There has been considerable recent discussion of methods for mitigating this bias through joint modeling. See Cook and Lawless (2021), who consider joint modeling of the disease and visits processes and Cook et al. (2022) who use joint modeling to accommodate a marker-dependent visits processes; they also provide references for related recent work.

Another issue concerning followup is dropout or loss to followup of cohort members. In this case so-called independent censoring assumptions are needed for transportability of study results. As with enrolment in a cohort, independent censoring cannot be guaranteed based only on cohort data. We have recently discussed the need for auxiliary data from tracing studies of persons lost to followup in order to address this (Lawless & Cook, 2019).

Under some assumptions, the likelihood functions given in Sect. 3.1 allow disease process models to be fitted. If independent delayed entry to the cohort is assumed, then $L_F = L_{R} L_P$ allows estimation of the process transition intensities, including observed covariate effects. However, if independence cannot be assumed, we are able to estimate intensities for persons in the study cohort using $L_P$ as in (3.3) but we cannot estimate intensities for persons outside the cohort: $L_R$ in (3.2) is a function of these intensities and of study recruitment intensities, but the separate intensities are non-identifiable. Cook and Lawless (2024b) demonstrate this for the process in Fig. 2; they also show how auxiliary data can be used to overcome the non-identifiability. We discuss some sources and uses of auxiliary information next.

## Other sources of information on disease processes

**Fig. 3:**
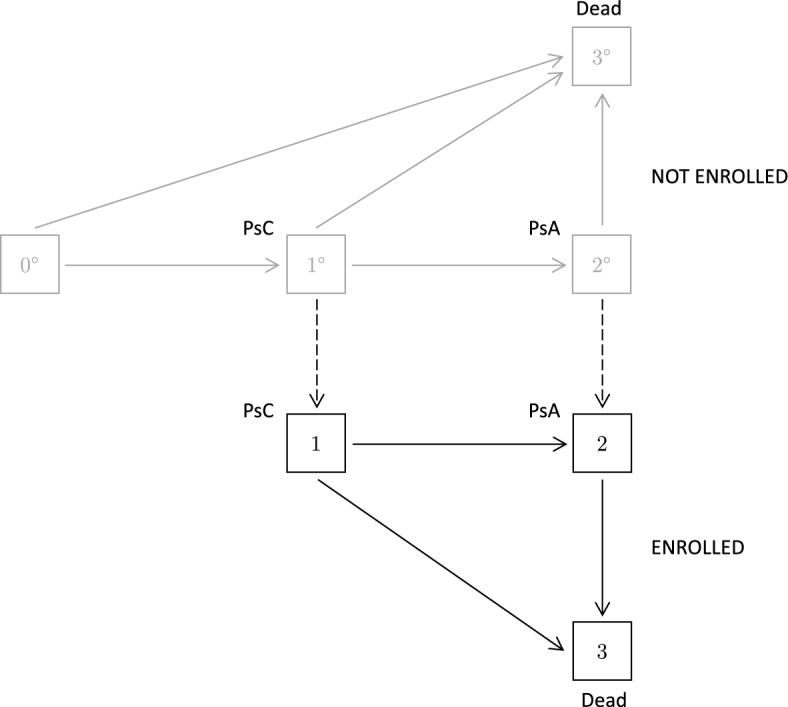
A joint model for PsC, PsA, death and study entry

We consider here some sources of information on disease processes that might be used in conjunction with a cohort study. To make the discussion concrete we consider the 4-state version of the process in Fig. 1, where state 1 corresponds to the diagnosis of some condition, state 2 represents some type of progression, and state 3 is death. We refer for illustration to the setting of Fig. 3, where states 1 and 2 are occupied by persons with psoriasis (Ps, also denoted as PsC) and psoriatic arthritis (PsA) respectively. Intensities of interest include those for onset of Ps and of PsA, and the death intensities from each of states 0, 1 and 2. We focus on the population of persons diagnosed with psoriasis and having entered state $1^{\circ }$. We consider a type B study in which cohort members are recruited from states $1^{\circ }$ or $2^{\circ }$; this can be represented by the process shown in Fig. 3. As suggested by Fig. 2, the transition intensities prior to and after enrolment in the cohorts may differ. The intensities for enrolment in the cohort from states $1^{\circ }$ and $2^{\circ }$ are of the form $\rho _1(t|{{\mathcal {H}}}(t))$ and $\rho _2(t|{{\mathcal {H}}}(t))$ respectively; for simplicity we often merely denote them as $\rho _1,\rho _2$. We consider analysis of this process in Sect. 6; here we describe other potential sources of information about it.

Sources of information on disease processes include electronic health records (EHR) created within health care systems, registries of persons with a specific disease in some population, surveys that determine disease status or history for a random sample of persons in a population, and aggregate data on disease incidence and mortality rates in a population over time. Such sources could in conjunction with a retrospective likelihood from a cohort study facilitate estimation of disease process features in the population or allow comparison of estimates based on the auxiliary data with estimates based on the likelihood $L_P$ for the prospective cohort data. An initial question, of course, is to what extent a data source is aligned with the study population from which a cohort is recruited. It should also be recognized that the quality and accuracy of data recorded can vary greatly across different types of data, and within different sources of the same type (e.g. Herrett et al., 2013; Togo & Yonemoto, 2022). We comment briefly on some of these sources.

### Electronic health records

Electronic health records (EHRs) for individual patients are maintained by providers of health services, including hospitals and other treatment facilities, public or private health insurance plans, and health management systems. These can represent persons with a disease process of interest, and supply longitudinal data on disease events and interventions. EHR data are frequently related to billing or payment for services and may not provide detailed or accurate information on a person’s medical condition. Numerous authors have discussed issues concerning data quality, and the fields of health and medical informatics have expanded efforts to deal with them in recent years. Common problems include selective populations (e.g. insured persons) or selective representation of a population (e.g. less well persons are over-represented) and selective data collection (again, biased towards less well persons); see for example Harton et al. (2022) and Hubbard et al. (2021). Important covariates and risk factors, including confounders related to treatment, may not be included, and missing data are common for variables that are present. There may be measurement or diagnostic error associated with disease outcomes, associated events and covariates (e.g. see Beesley & Mukherjee, 2022; Eder et al., 2019); this may be related to the systems of coding and data entry used for databases. The assembly of a data set for statistical analysis is often difficult, and to get crucial factors on individuals it is necessary to link separate databases. Records that are related to treatment in facilities such as hospitals or clinics may have much more detailed information, though missing data and measurement error is common here too (e.g. Ieva et al., 2017).

EHR data are often used for individual studies, or to provide specific data (e.g. prescription information, test results) on individuals who are members of a cohort. In the former case they can provide a prospective likelihood as in (3.3); as in a cohort study, it is important in this case to account for selection conditions associated with the database (e.g. Beesley & Mukherjee, 2022). When used for comparison with results from a cohort study, EHR data are often limited since they typically do not include certain variables measured within the cohort. It is also possible to use EHR databases to estimate the prevalence and incidence of various diseases in specific populations; this can be useful in assessing results from cohort studies. The accuracy of disease identification based on algorithms applied to the coding systems in an EHR database should be carefully assessed. Eder et al. (2019) provides an illustration concerning Ps and PsA in Ontario, Canada.

### Disease registries

For certain diseases or conditions, persons in a population may be registered in a database that includes the date of disease onset, demographic factors (e.g. age, sex) and other information. Subsequent related information may also be added over time; for example, registries of persons with specific forms of cancer may later add dates and causes of death. The quality of registry data varies widely with respect to the amount of information and its completeness and accuracy. The SEER cancer registry of the US National Cancer Institute (see http://seer.cancer.gov) includes data on cancer patients diagnosed since 1973, and provides survival rates and other statistics stratified by age, sex, and year and age of diagnosis. More specialized registries, for example of persons with chronic kidney disease in the Swedish Renal Registry (Rehnberg et al., 2024) and of persons waitlisted and receiving organ transplants (e.g. the U.S. Scientific Registry of Transplant Recipients, see https://www.srtr.org) provide comprehensive data but checks on accuracy and completeness (e.g. Rehnberg et al., 2024) are recommended.

Registries can serve several functions relative to cohort studies. In some settings a cohort study might recruit participants from members of a registry. This would ideally be done according to a (stratified) random sampling scheme, so that independent recruitment and cohort entry conditions are satisfied. The combined registry and cohort data would together comprise what is known as a two-phase study (see Section 7.2 of Cook & Lawless, 2018); the registry is phase 1 and the cohort is referred to as the phase 2 study. In the settings we consider here, selection probabilities for the phase 2 study would take the form

4.6 $$\begin{aligned} \pi _i = P( \Delta _i = 1 | H^{\textrm{reg}}_i(l), L_i = l), \end{aligned}$$

where $\Delta _i=1,0$ indicates whether person *i* in the registry was selected or not selected for phase 2, respectively, $L_i=l$ is the time at which they are selected (or not), and $H^{\textrm{reg}}_i(l)$ is the registry information on which the selection is based. Followup and observation of individuals in the cohort allows for the collection of data on events and covariates that are not part of the registry records. Cook and Lawless (2018) describe methods of estimation for two-phase disease history studies.

Another use of registries arises when there is a type B cohort where enrolment is at least in part individual-driven, but where all persons with the condition of interest are included in a registry. In this case we can, at a given time, identify who has enrolled in the cohort and who has not. This provides a likelihood function based on this information which, in conjunction with the restrospective likelihood based on persons in the cohort, can allow estimation of cohort entry intensities (Cook & Lawless, 2024b). We emphasize that the likelihood based on enrolment status needs to recognize disease features available in the registry data. The probabilities $\pi$ for enrolment in the cohort by a given time $L_i=l$ for individual *i* are of the same general form as (4.6). When individuals in the registry are independent as far as cohort enrolment is concerned, this can provide a product binomial likelihood function with terms of the form

4.7 $$\begin{aligned} L_{Bi} = P( \Delta _i | H^{\textrm{reg}}_i(l), L_i = l), \end{aligned}$$

where $\Delta _i=1$ and 0 respectively indicate enrolment and non-enrolment in the cohort by a given time $L_i=l$. It is important in this setting that $H^{\textrm{reg}}$ include information on those factors which influence enrolment in the cohort. This is not always the case.

In settings where cohort members are not necessarily in a registry but the registry and cohort populations are considered similar, a third use of registry data is in providing estimates of specific disease features which may be compared with estimates of those features based on the cohort study. If variables affecting cohort enrolment are not available in the registry data, then we may expect to see differences in the estimates from the two sources. Interpretation and comparison of the separate estimates may then be difficult; for the registry this can be due to unknown effects of variables not recorded in the registry, and for the cohort, for unknown factors affecting enrolment. In general, features being compared will typically be marginal features that involve averaging over factors not available in the registry data, and the distribution of these unmeasured factors in the registry and cohort may differ because of enrolment conditions.

Finally, there has been much current interest in using registries and other sources as auxiliary data that can be combined with cohort data, and thereby improve efficiency of estimation (e.g. Huang et al., 2016; Shi et al., 2021). This requires strong assumptions; specifically, disease process intensities must be similar in the the cohort and in the auxiliary data populations, conditional on important covariates. Huang et al. (2016) provide an illustration concerning advanced prostate cancer where they use estimates of five year survival rates (from diagnosis) stratified by certain factors based on the SEER cancer registry (http://seer.cancer.gov) to augment data from a cohort.

### Health surveys

Longitudinal or cross-sectional health surveys based on a random sample from a population are another source of information on disease. Some longitudinal surveys, such as the Canadian Longitudinal Survey on Aging (see https://www.clsa-elcv.ca) and the US National Health and Nutrition Examination Survey (see https://www.cdc.gov/nhanes) have a broad mandate. Data may be collected for individuals in the survey on specific conditions or diseases at successive observation times, but the level of detail is usually less than for cohort studies or surveys focused on a specific disease. Longitudinal surveys can however be regarded as cohort studies with the benefit of selection of individuals based on random sampling of a population (perhaps stratified and cluster-based). Even with rather little detail on covariates and other factors, and with intermittent collection of data at times that are more widely spaced than in cohort studies, they may be able to provide stratified estimates of disease prevalence and incidence.

Cross-sectional surveys (perhaps repeated over time) can provide estimates of disease prevalence, and possibly of incidence if accurate data on disease diagnosis times for sample members are obtained. This might also provide limited retrospective data on disease progression. A series of annual cross-sectional surveys on psoriasis and psoriatic arthritis are conducted by the US National Psoriasis Foundation (https://psoriasis.org/annual-survey/).

### Disease incidence and mortality rates

For some diseases there are registries that provide population incidence, prevalence and mortality statistics. The SEER cancer registry mentioned in Sect. 4.2 is an example. Other population level disease rates are provided by government agencies; in Canada, Statistics Canada (https://www.statcan.ca/) maintains records of mortality rates and cancer-related rates over time. In the United States, the National Center for Health Statistics at the Centers for Disease Control and Prevention (https://www.cdc.gov/nchs/products/) provides information on a wide range of medical conditions. These sources can provide estimates, for example of prevalence or mortality rates, that may be compared with similar estimates based on a disease cohort. As noted in Sect. 4.2, there is also much interest in using data or estimates from such sources to augment inferences from cohort studies. Shi et al. (2021) provide an illustration where they use population mortality data to augment the analysis of mortality in a cohort of US nuns. Many cohort members were born long ago and it was important to stratify mortality rates according to sex, age and year of birth.

## Assessing interventions

Treatment or management of a disease typically involves therapeutic interventions which have been assessed in randomized control trials (RCTs) and given regulatory approval. However, estimated effects from such trials do not correspond to effects on individual patients; recent interest in personalized and stratified medicine comes from a desire to estimate treatment effects for individuals or relatively homogenous groups of individuals. This involves adjusting for conditions specific to the individual or group of interest. Due to narrow inclusion criteria, effects from clinical trials are also not generalizable to broad populations of patients—interest in “real world” effects manifest in actual populations (e.g. Crown, 2019; Togo & Yonemoto, 2022) arises from the need for more realistic measures of population impact. Information is available from cohort studies, but because therapies are assigned according to a person’s current condition and disease history (i.e. “by indication") and other factors such as medical coverage, an objective assessment of the effects of therapies is challenging. There has been considerable recent research in this area. Much effort has been directed at causal inferences regarding treatment effects that align with those available in RCTs (e.g. Crown, 2019) but such goals are very challenging. One approach concerns estimation of an average causal effect (ACE) based on an average treatment effect (ATE) from observational data by emulating a setting where treatments are randomly assigned to each person. This is often done using propensity score weighting or matching. Because therapies are assigned dynamically in real world cohorts and may be initiated or terminated at arbitrary times, it is necessary to consider multiple time intervals and to aggregate estimates over multiple intervals (e.g. Dickerman et al., 2019). Proposed methods involve assumptions about stability of treatment effects over time, the need for study participants at any given time to have a positive probability of being on alternative treatments and the need for correct models for treatment assignment. Such methods, and related use of counterfactual outcomes, do not deal well with the dynamic aspects of disease and therapeutic interventions, including treatment initiation or changes at arbitrary times, and because they involve averages over complex time-varying factors, interpretation is difficult.

Our view is that the concept of an ATE is of limited relevance when considering the treatment of individual patients in clinical settings. When individual–level risk factors are unavailable for a patient belonging to a comparable population to the one for which the ATE is computed, or if there is little information about heterogeneity of treatment effects, it can guide treatment decisions. However, these are rather rare occasions for well-studied disease processes and established treatments. In general an ATE, defined within a conceptual framework wherein treatments are randomly assigned to patients (as opposed to according to their individual condition), does not correspond to how interventions are delivered in the clinical care settings, and such an ATE will not reflect the anticipated benefit for a specific individual.

Other approaches adopt models in which the effects of a therapy are conditional on disease-related factors that may influence choice of therapy. We argue for the use of such models but in an expanded framework that simultaneously considers the disease process, related covariate processes and the treatment process, all of which are dynamic. In most observational cohorts the data collection process also needs careful consideration, since “observation” of a patient often is disease-related. Such models provide insight into treatment assignment and treatment effects, which can be studied through predictive models for specific disease outcomes. This is aligned with real world clinical practice in which dynamic predictive models concerning the risks of future events are used when counselling patients and recommending therapies. This does not, however, produce causal effects in the sense they are considered in RCTs, but it is aligned with the concept of dynamic or Granger causality (e.g. Aalen et al., 2008). Cohort studies that involve rigorous collection of data on a diverse set of participants are especially valuable. This facilitates modeling of disease processes that utilizes both fixed covariates and time-varying factors such as biomarkers, treatment and prior disease history. In addition, factors associated with initiation, changes or termination of treatment can be studied. We now describe this in a little more detail.

Let *Z*(*t*) denote the multistate disease process, *X*(*t*) represent a vector of time-varying covariates (e.g. biomarkers), *B*(*t*) a time-varying indicator of receiving treatment, and *V* a vector of fixed covariates. Let ${{\mathcal {Z}}}(t) = (Z(t), X(t), B(t))$ and $\mathcal{H}(t) = \{ {{\mathcal {Z}}}(s), 0 \le s< t, V\}$. Our approach to analysis is to formulate disease process models that express how covariates, treatment and disease history affect changes in disease status. This often involves multistate models with transition intensities $\lambda _{kr}(t \mid {{\mathcal {H}}}(t))=\lambda _{kr0}(t |{{\mathcal {H}}}(t)) \, g(X(t), B(t), V; \beta )$ where the function $\lambda _{kr0}(t | \mathcal{H}(t))$ is a baseline intensity governing $k \rightarrow r$ transitions that may depend on the disease process history ${H}(t) = \{Z(s), 0\le s< t\}$, and $g(\cdot ;\beta )>1$ is a multiplicative term modulating this intensity. Note that both *X*(*t*) and *B*(*t*) may be defined as the current value of markers and treatment, or to be functions of previous and current covariate values and treatment histories respectively. We consider some particular models in the next section.

When *X*(*t*) or *B*(*t*) is under intermittent observation, a joint modeling approach is needed and we illustrate such a model in Sect. 6 when modeling the effect of biologics therapy on the development of arthritis mutilans in patients with psoriatic arthritis. Fitting a model for a process that incorporates the “event" of biologic treatment initiation allows computation of the probability of disease progression by time $t+s$, conditional on covariates at time *t*. The treatment represented by *B*(*t*) is said to have an effect, in the Granger sense of causality, if it significantly improves the predictive power of the model (Eichler & Didelez, 2010; Granger, 1988). Using such a joint model we can also compare the probability of disease progression by time $t+s$ for individuals with $B(t)=1$ and $B(t)=0$ respectively; this is similar to an intention to treat comparison in a RCT, since a person with $B(t)=0$ could start treatment some time between *t* and $t+s$. We note two points: First, such inferences are highly model dependent, so assessment of model assumptions is crucial. Second, the model formulation is best guided by context-specific knowledge of the processes under study, since the models themselves cannot provide clear insights into causal pathways concerning markers, treatment and disease. Insight can be gained however from careful examination of the component intensities and more generally, of factors affecting them. Specifically, the treatment initiation intensities characterize the treatment policy adopted in the cohort. In general there are random elements associated with timing, physicians’ views, treatment availability and patient consent, and the intensities allow for this. In many settings treatment policies and coverage involve rules that exclude some patients at any given time *t*, in which case the treatment initiation intensity at time *t* may be zero for certain disease and covariate histories.

## Illustrative analyses involving psoriatic arthritis

We consider here some inferences based on data from the psoriasis cohort UTPC and the psoriatic arthritis cohort UTPAC described in Sect. 1. We will consider two features: (i) the risk of psoriatic arthritis (PsA) following onset of psoriasis (Ps) and (ii) the risk of disease progression from PsA to PsA with arthritis mutilans, defined here as the accumulation of three or more damaged joints. The combined disease process can in this case be represented by the case $K=3$ in Fig. 1, with state 1 entered upon the diagnosis of Ps, state 2 entered upon the diagnosis of PsA and state 3 entered when a third damaged joint is identified. The enrolment process for the combined cohorts is represented in Fig. 3; we assume for simplicity here that persons have fewer than three damaged joints at the time of PsA diagnosis.

Our first objective is to study the risk of PsA among persons with Ps, which involves the intensity for transitions from state 1 to state 2 of Fig. 3. We assume that the visit process is independent of the disease process but comment later on this assumption. Based on data from prospective followup of persons enrolled in the UTPC, the prospective likelihood $L_P$ is given by (3.3), with $L=l$ the time of enrolment (measured in terms of time since Ps onset) in the UTPC and $Z(l)=1$. The onset time for Ps and hence the time *L* of enrolment are assumed known. We do not consider time-varying covariates or treatment in these analyses but let the vector *V* contain fixed covariates including age at Ps onset centered at 40 years ($V_{1}$), sex with 1 for male and 0 for female ($V_{2}$), and human leukocyte antigen (HLA) B27, a genetic marker that has been found to be important in PsA, with 1 denoting HLA B27 positive and zero denoting negative ($V_{3}$). The analyses described here were conducted using the R package msm() (Jackson 2010), which handles intermittent observation at irregular clinic visit times.

Table 1 shows selected results from analyses in Cook and Lawless (2024b). Estimates of the log baseline intensity and regression coefficients from the multiplicative intensity model with $\lambda _{12}(t|H(t))= \lambda _{12} \exp (\theta ' V)$ are reported along with estimates and 95% confidence intervals for the exponentiated coefficients (relative risks, RR). The estimated baseline intensity $\lambda _{12}$ for PsA is small and there is quite high variability in the estimates of the covariate effects for $V_2$ and $V_3$, in part because a relatively small proportion of individuals (61 of 715) were observed to make the $1 \rightarrow 2$ transition. Using the estimates shown, and under the assumption that mortality rates are negligible, we can calculate that the risk of developing PsA by age 60 for an HLA-B27 negative male aged 40 at Ps onset is 0.25; for a corresponding female it is 0.22. More extensive analyses are possible; for example we can easily consider non-homogeneous baseline intensities and additional covariates. It is also possible to use retrospective information on the time of PsA diagnosis for persons in the UTPAC cohort who were recruited after diagnosis of PsA, making the assumption that the intensity for PsA onset is the same pre-enrolment in the cohorts as it is after enrolment in UTPC. Cook and Lawless (2024b) provide details.

**Table 1:** Estimates from regression modeling for the development of PsA in psoriasis patients based on data from the UTPC registry with covariates including age of onset of psoriasis (years) centered by 40 ($V_1$), sex ($V_2$), and HLA B27 status ($V_3$)

Covariate	Parameter	Est.	s.e	RR	95% CI	p-value
Baseline intensity	$\log \lambda _{12}$	$-$4.379	0.250			
Age of onset for Ps (centered at 40)	$\theta _{1}$	$-$0.011	0.009	0.99	(0.97, 1.01)	0.255
Sex (1 = male, 0 = female)	$\theta _{2}$	0.117	0.298	1.12	(0.63, 2.01)	0.695
HLA-B27 (1 = positive, 0 = negative)	$\theta _{3}$	$-$0.724	1.012	0.48	(0.07, 3.52)	0.474

**Fig. 4:**
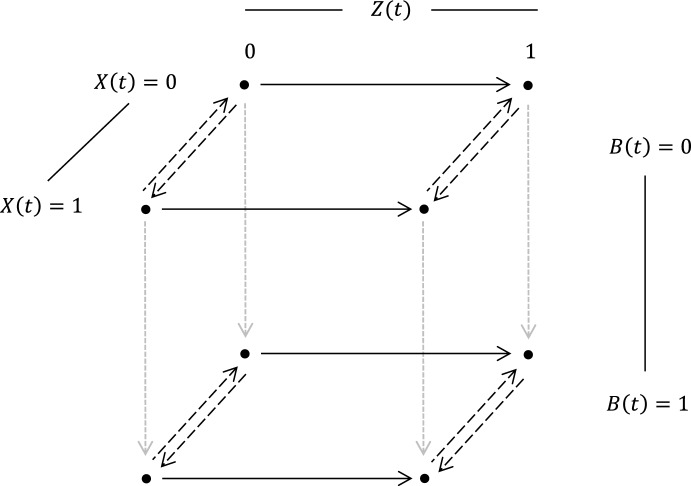
A joint model for a dynamic binary marker, treatment status and disease progression

We next consider persons with PsA and the risk of developing arthritis mutilians defined by the presence of three or more damaged joints. We restrict attention to individuals in UTPAC who have zero damaged joints at their first clinic visit following January 1, 2000 (the year biologics were introduced) and consider time of this visit as $t=0$. We let *T* be the time that arthritis mutilans developed and define $Z(t) = I(T \le t)$ so that $Z(t)=1$ for an individual with arthritis mutilans by time *t* and $Z(t)=0$ otherwise. The erythrocyte sedimentation rate (ESR) is a dynamic inflammatory marker known to be associated with disease activity. We dichotomize it here using established thresholds defining normal levels as $\le$15 mm/h and $\le$ 20 mm/h for males and females under 50 years of age respectively; we thus use a binary time-dependent marker with $X(t)=1$ indicating an inflammatory state and $X(t)=0$ a normal state. If *B* is the time biologics are prescribed we let $B(t) = I(B \le t)$ indicate that a prescription has been filled by time $t>0$; we restrict attention to persons who are initially not receiving treatment and once a person initiates treatment assume they remain on it. When considering the failure, marker and treatment processes jointly we can define an 8-state process with states labeled by the triple of indicators ${{\mathcal {Z}}}(t) =(Z(t),X(t),B(t))$. Figure 4 portrays the joint failure-marker-treatment process. We let $a_0$ denote the initial visit time; the initial conditions mean that individuals start in states (0, 0, 0) or (0, 1, 0) at $a_0$. Transitions to the right represent the development of arthritis mutilans; the four arrows convey the fact that the risk of arthritis mutilans can depend on the marker and treatment status. Downward transitions occur upon treatment initiation and these may depend on the marker and failure status. Transitions toward the front or back set of states occur as the marker value changes. Here fixed covariates include indicators of having 3–4 ($V_{1}$) or $\ge 5$ ($V_{2}$) swollen joints at the first visit, and an indicator of whether the disease duration was greater than 5 years at the time of the first visit ($V_{3}$).

Our interest is in the effect of treatment and biomarker status on risk of arthritis mutilans. We therefore consider the failure intensity function

6.1 $$\begin{aligned} \lambda ^z(t \mid B(t), \mathcal {H}(t))=\lambda ^z_\circ (t) \exp (X(t) \beta _1 + B(t) \beta _2 + X(t)B(t) \beta _3 + V'\beta _3), \end{aligned}$$

where $\lambda ^z_\circ (t)$ is piecewise constant with cut-points at 5 and 10 years from January 1, 2000 to accommodate possible time-trends related to biologics availability. For the marker process we write the two transition intensities as $\lambda ^x_{k}(t \mid B(t), {{\mathcal {H}}}(t))$ as

6.2 $$\begin{aligned} \lim _{\Delta t \downarrow 0} \dfrac{\textrm{P}(X(t + \Delta t^-) = k \mid X(t^-) = 1-k, B(t), {{\mathcal {H}}}(t))}{\Delta t} = \lambda ^x_{k\circ }(t) \, \exp (B(t)\eta _{k1} + V'\eta _{k2}), \nonumber \\ \end{aligned}$$

for $k=0, 1$ where $k=0$ applies to the $1 \rightarrow 0$ transition and $k=1$ to the $0 \rightarrow 1$ transition, and the baseline intensities are piecewise constant with the same cut-points as the failure intensity. The intensity for the prescription of biologic therapy $\lambda ^b(t \mid B(t^-)=0, \mathcal {H}(t))$ is

6.3 $$\begin{aligned} \lim _{\Delta t \downarrow 0}\frac{P(B(t+\Delta t^-)=1 \mid B(t^-)=0, {{\mathcal {H}}}(t))}{\Delta t} =\lambda ^b_\circ (t) \exp (X(t^-) \phi _1 + V' \phi _2); \end{aligned}$$

Individuals are under intermittent observation and we let $a_{i0}, a_{i1}, \ldots , a_{im_i}$ denote the $m_i$ assessment times for individual *i* and let $A_i(t)$ count the number of visits over (0, *t*]. We assume in what follows that the visit process is conditionally independent of the joint process (Cook & Lawless, 2021) and fit the 8-state model to data of the form $\{({{\mathcal {Z}}}_i(a_{ij}), a_{ij}), j=1,\ldots , m_i, V_i\}$ based on the likelihood below to draw inferences regarding the three processes. We let ${{\mathcal {H}}}_i^{\textrm{obs}}(t) = \{ (\mathcal{Z}_i(a_{ij}), a_{ij}), j=0,1,\ldots , A_i(t^-), V_i\}$ denote the observed history at time *t*. With a sample of *n* independent processes, the likelihood is

6.4 $$\begin{aligned} L \propto \prod _{i=1}^n \prod _{j=1}^{m_i} P ({{\mathcal {Z}}}_i(a_{ij})| a_{ij}, {{\mathcal {H}}}^{\textrm{obs}}_i(a^-_{ij}) ) \end{aligned}$$

We also considered a model incorporating information recorded at clinic visits on the number of swollen joints instead of using just baseline values. Specifically we define $V_{i1}(t) = V_{i1}(a_{iA_i(t^-)})$ and $V_{i2}(t) = V_{i2}(a_{iA_i(t^-)})$ as the time-dependent indicators of 3–4 or $\ge 5$ swollen joints at the most recent assessment. If we let $V(t) = (V_1(t), V_2(t), V_3)'$ where $V_3$ is defined as earlier, then the intensities in (6.1)-(6.3) can be revised by replacing *V* with *V*(*t*). If the history is redefined as ${{\mathcal {H}}}_{i}^{\textrm{obs}}(t) = \{ (\mathcal{Z}_i(a_{ij}), V_i(a_{ij}), a_{ij}), j=0,1,\ldots , A_i(t^-)\}$ the modified likelihood incorporating the covariates defined at the most recent clinic visit is still based on (6.4). We report the results from fitting this model in Table 2.

**Table 2:** Estimates from fitting the joint model depicted in Fig. 4 with the time-dependent covariates based on the total number of swollen joints ($(V_{1}(t), V_{2}(t))'$) and fixed covariate ($V_3$) indicating a disease history of more than 5 years at the first visit

Process	Transition	Covariate	Est.	s.e	RR	95% CI
Marker	$1 \rightarrow 0$	Biologics, *B*(*t*)	0.335	0.101	1.40	(1.15, 1.70)
		3–4 Swollen Joints, $V_{1}(t)$	0.686	0.419	1.99	(0.87, 4.51)
		$\ge 5$ Swollen Joints, $V_{2}(t)$	$-$0.074	0.273	0.93	(0.54, 1.59)
	$0 \rightarrow 1$	Biologics, *B*(*t*)	0.120	0.101	1.13	(0.93, 1.37)
		3–4 Swollen Joints, $V_{1}(t)$	0.813	0.461	2.25	(0.91, 5.57)
		$\ge 5$ Swollen Joints, $V_{2}(t)$	0.157	0.333	1.17	(0.61, 2.25)
Biologics	$0 \rightarrow 1$	Elevated ESR$^\dagger$, *X*(*t*)	0.618	0.197	1.86	(1.26, 2.73)
		3–4 Swollen Joints, $V_{1}(t)$	0.347	0.393	1.42	(0.65, 3.06)
		$\ge 5$ Swollen Joints, $V_{2}(t)$	1.004	0.282	2.73	(1.57, 4.75)
Failure	$0 \rightarrow 1$	Elevated ESR$^\dagger$, *X*(*t*)	0.554	0.399	1.74	(0.80, 3.80)
		Biologics, *B*(*t*)	1.081	0.297	2.95	(1.65, 5.27)
		*X*(*t*)*B*(*t*)	$-$1.657	0.732	0.19	(0.05, 0.80)
		3–4 Swollen Joints, $V_{1}(t)$	0.327	0.528	1.39	(0.49, 3.91)
		$\ge 5$ Swollen Joints, $V_{2}(t)$	1.334	0.360	3.79	(1.87, 7.69)
		$\ge 5$ years PSA, $V_3$	0.117	0.217	1.12	(0.74, 1.72)

$^\dagger$ When ESR was unavailable, classification was based on C-reactive protein levels

For the marker process we see that biologic therapy has a strong and significant effect on increasing the $1 \rightarrow 0$ intensity ($RR = 1.4$). There is no significant evidence of an association between swollen joint count and changes in marker status, but confidence intervals for the relative risks are rather wide. Biologics are prescribed at a much higher rate to individuals with elevated marker ($RR=1.86$), as well as those with at least 5 damaged joints; having at least five swollen joints was required at one point for insurance coverage of biologic therapy. For the failure process we see that an elevation in the inflammatory marker $(X(t)=1)$ increases the risk of developing arthritis mutilans ($RR=1.74$), as does the presence of at least 5 swollen joints. Among individuals with an elevated marker status when controlling for the number of swollen joints at the last visit and disease duration (i.e. *V*(*t*)), biologic therapy reduces the risk of progression to arthritis mutilans ($RR=0.56$; 95% CI: 0.17, 1.88). Given *V*(*t*), there is evidence of an increased risk of arthritis mutilans ($RR=2.95$) among individuals with a normal inflammatory marker $(X(t)=0)$; this is likely the result of unaccounted for dynamic confounders. The assumption of a conditionally independent visit process may also be violated and therefore a source of bias. This can be mitigated by fitting joint models for the process of interest (here $\{ {{\mathcal {Z}}}(s), 0 \le s\}$) and the visit process $\{ A(s), 0< s\}$ (Cook & Lawless, 2021; Lange et al., 2015). In simpler analyses involving data from the UTPAC we have found some evidence of dependence of the visit process on the disease and marker processes, but this is beyond the scope of the current manuscript. We remark also that model checks showed good agreement between the model-based estimate of the probability of failure by time *t* and a corresponding nonparametric estimate. Further research into model assessment with complex processes as we employ here is warranted, but beyond our present scope.

## Concluding remarks

Health scientists seldom have representative samples on which to base analyses and it can be very challenging to address selection effects when little information is available on the study enrolment process. We have discussed various sources of information which can help gauge representativeness of the available sample, but addressing selection biases when they arise from dynamic aspects of disease processes or care can be difficult. While it is generally important to understand how the sample relates to a possible target population of processes to ensure broad validity, some disease features are affected by selection effects more than others. Covariate effects expressed in rich intensity-based models may be less affected, for example, than predictions which depend on both estimated intensities and covariate effects. Further work is warranted to help understand which features of dynamic processes are most susceptible to biased selection schemes and other types of biased observation.

We have separately considered the issues of selection effects, dependent repeated observation, and assessment of treatment effects when there is confounding by indication, but these often arise together when patients attend tertiary care clinics based their underlying condition and receive stronger therapies as a result; the joint effects of these biases can also be more pronounced in the analysis of electronic medical records. The ability to address these biases depends critically on the availability of key information. In cohorts maintained by clinician scientists it is important to record as much information as possible on the disease histories prior to enrollment, as well as the way in which patients were identified. When data are collected at clinic visits it is likewise important to record whether visits were scheduled as part of a protocol or whether they were prompted by worsening health and the need for additional healthcare. Information on loss to followup is often lacking since participants may simply discontinue engagement in a registry-based cohort without declaring a withdrawal date. In this case tracing studies (Lawless & Cook, 2019) can be helpful to both clarify whether patients should be viewed as withdrawn and to solicit information on current health status. Finally, recording reasons for the prescription, switching, or discontinuation of treatment will support more detailed modeling of the treatment process.

In principle, joint modeling of selection, observation (visits and loss to followup) processes, and treatment assignment can be carried out to mitigate biases but there may be settings where a combination of different approaches to bias mitigation could be appealing. Here one can explore the robustness and relative efficiency of different mitigation strategies and how they may be used together most effectively. There has been much interest in recent years in the use of inverse intensity weighting (Lin et al., 2004; Pullenayegum, 2020) for dealing with covariate or disease related intermittent visits and Coulombe et al. (2021) consider this in concert with weights based on the inverse probability of treatment. An appealing feature of joint modeling is that observation and treatment processes are studied along with the disease process. Moreover, inferences are based on models with transparent assumptions which can be checked for plausibility based on observed data.

## Data Availability

The data on which the analyses in Sect. 6 are based are protected by a patient confidentiality agreement and are not available for dissemination.
